# Functional Connectivity Between the Trigeminal Main Sensory Nucleus and the Trigeminal Motor Nucleus

**DOI:** 10.3389/fncel.2020.00167

**Published:** 2020-06-23

**Authors:** Mohammed Slaoui Hasnaoui, Isabel Arsenault, Dorly Verdier, Sami Obeid, Arlette Kolta

**Affiliations:** ^1^Groupe de Recherche sur le Systéme Nerveux Central, Département de Neurosciences, Faculté de Médecine, Université de Montréal, Montréeal, QC, Canada; ^2^Département de Stomatologie, Faculté de Médecine Dentaire, Université de Montreal, Montreal, QC, Canada

**Keywords:** mastication, trigeminal, NVsnpr, NVmt, motoneurons, jaw muscles, burst firing

## Abstract

The present study shows new evidence of functional connectivity between the trigeminal main sensory (NVsnpr) and motor (NVmt) nuclei in rats and mice. NVsnpr neurons projecting to NVmt are most highly concentrated in its dorsal half. Their electrical stimulation induced multiphasic excitatory synaptic responses in trigeminal MNs and evoked calcium responses mainly in the jaw-closing region of NVmt. Induction of rhythmic bursting in NVsnpr neurons by local applications of BAPTA also elicited rhythmic firing or clustering of postsynaptic potentials in trigeminal motoneurons, further emphasizing the functional relationship between these two nuclei in terms of rhythm transmission. Biocytin injections in both nuclei and calcium-imaging in one of the two nuclei during electrical stimulation of the other revealed a specific pattern of connectivity between the two nuclei, which organization seemed to critically depend on the dorsoventral location of the stimulation site within NVsnpr with the most dorsal areas of NVsnpr projecting to the dorsolateral region of NVmt and intermediate areas projecting to ventromedial NVmt. This study confirms and develops earlier experiments by exploring the physiological nature and functional topography of the connectivity between NVsnpr and NVmt that was demonstrated in the past with neuroanatomical techniques.

## Introduction

Mastication is a vital behavior that allows the preparation of food for swallowing during feeding. This rhythmic movement can be initiated by repetitive stimulation of either the cortical masticatory area (CMA) or the trigeminal sensory afferents while its pattern of activity is shaped by a neuronal network in the brainstem known as the masticatory central pattern generator (CPG; Bremer, 1923; Dellow and Lund, 1971). Combined evidence from several *in vitro* studies suggests that the CPG is located in the pons near the trigeminal motor nucleus (NVmt; Kogo et al., 1996; Nakamura et al., 1999; Tanaka et al., 1999).

In rodents, motoneurons (MNs) innervating masticatory muscles are clustered in two distinct divisions in the NVmt, a large dorsolateral (DL) and a much smaller ventromedial (VM) containing respectively the jaw-closing and jaw opening MNs (Mizuno et al., 1975; Limwongse and DeSantis, 1977; Sasamoto, 1979; Jacquin et al., 1983; Lynch, 1985; Rokx and van Willigen, 1985). The DL division extends throughout the entire rostrocaudal length of the NVmt, while the VM division is limited to its caudal two thirds. Masticatory muscles are often categorized as jaw opening and jaw-closing muscles, but many of them are subdivided in neuromuscular compartments allowing for the production of complex movements. These compartments represent the smallest anatomical muscular division which activation generates a unique movement (Widmer and Morris-Wiman, 2010). They are innervated independently and can be recruited either individually or in a group by the CPG. For instance, the rabbit masseter muscle was shown to be composed of at least 23 different neuromuscular compartments each innervated by axons of a single motor unit (English et al., 1999; Widmer et al., 2003). Thus, the differential recruitment of these compartments by the trigeminal pre-MNs would allow the production of diverse and precise jaw movements. This implies a much more complex myotopic organization of the NVmt paralleled by a functional topographical organization of the projections from the masticatory CPG.

Several lines of evidence suggest that the trigeminal main sensory nucleus (NVsnpr), one of the regions near NVmt, is likely to play a crucial role in either pattern and/or rhythm generation, placing it at the very heart of the masticatory CPG.

NVsnpr receives inputs from the CMA in the rat and trigeminal sensory afferents in rats and cats which allow respectively the initiation and adaptation of masticatory movements (Yasui et al., 1985; Shigenaga et al., 1986a,b, 1988; Yoshida et al., 2009). Bouts of masticatory movements increase blood flow to the NVsnpr nucleus in humans (Viggiano et al., 2015) and even bouts of fictive mastication in rabbits are associated to increased neural activity, as detected by C-Fos in its dorsal part (Athanassiadis et al., 2005a), and rhythmic firing of many of its neurons in phase with either the closing or opening motoneurons (Tsuboi et al., 2003). Furthermore, these dorsal neurons were shown to have intrinsic bursting properties in gerbils (Sandler et al., 1998) and rats (Brocard et al., 2006). In rats, this intrinsic bursting relies on a sodium persistent current (I_NaP_) whose appearance coincides with the emergence of the first masticatory movements (Brocard et al., 2006). This voltage-dependent current is also sensitive to variations in extracellular Ca^2+^-concentration (Li and Hatton, 1996; Su et al., 2001; Brocard et al., 2006, 2013; Tsuruyama et al., 2013; Morquette et al., 2015), and we have shown that small local extracellular applications of the Ca^2+^-chelator BAPTA can trigger rhythmic firing in rats NVsnpr neurons.

Anatomical evidence in mice, rats, cats, and rabbits suggest that direct projections from NVsnpr to trigeminal MNs exist (Mizuno et al., 1983; Landgren et al., 1986; Li et al., 1993; Kolta et al., 2000) and in rats, these seem topographically organized with the dorsal and intermediate regions projecting respectively to the DL and VM regions of NVmt (Li et al., 1995). However, the size of tracer injections and the presence of fibers passing through the NVmt limit interpretation of the results of these anatomical studies. Besides these anatomical studies of connectivity, and an electrophysiological investigation led in newborn rats (Nonaka et al., 2012) little is known about the functional relationship between NVsnpr neurons and trigeminal MNs. Therefore, the purpose of this study was to investigate electrophysiologically functional connectivity between NVsnpr and NVmt in more mature rats and to verify whether similar anatomical and electrophysiological findings can be obtained in mice to validate the use of transgenic mice expressing a genetically encoded Ca^2+^-indicator (GECI) in motoneurons and NVsnpr neurons for mapping purposes.

Our results suggest that NVsnpr and NVmt are topographically connected in both rats and mice and that rhythmic firing in NVsnpr neurons can drive rhythmic activation of trigeminal MNs.

## Materials and Methods

All experiments were conducted according to the Canadian Institutes of Health Research rules and were approved by the Animal Care and Use Committee of Université de Montréal. Thirty-five Sprague–Dawley rats (Charles River, Montreal, QC, Canada) and 90 Thy1-GCaMP6f transgenic mice (C57BL/6J-Tg(Thy1-GCaMP6f)GP5.17Dkim/J, stock 025393, The Jackson Laboratory, Sacramento, CA, USA) were used in this study. The transgenic mice express a genetically encoded green fluorescent calcium indicator (GCaMP6f) used for imaging-based monitoring of neuronal activity in individual neurons (Chen et al., 2013).

### Retrograde Labeling of Trigeminal Motoneurons in Rats

Pups (2–5 days old) were first injected with 5–10 μl of the retrograde tracer Cholera Toxin conjugated with Alexa Fluor 488 (Molecular Probes, Eugene, OR, USA) into their digastric muscles under hypothermic anesthesia. Crystals of the carbocyanine dye 1.1′-dioctadecyl-3, 3,3′,3′-tetra-methylindocarbocyanine perchlorate DiI [DiIC_18_ (3), Molecular Probes, Eugene, OR, USA] were then inserted within their masseter muscles using the tip of a needle. The tracers were allowed several days to diffuse (9–28 days) before the animals were used for experiments.

### Brainstem Slice Preparations in Rats and Mice

Experiments were conducted on slices obtained from either the rats (aged P12–27) previously injected with the retrograde tracers or from Thy1-GCaMP6f mice (aged P8–26). The animals were anesthetized with isoflurane (Pharmaceutical Partners of Canada Inc., Richmond Hill, ON, Canada) before decapitation. Their brain was quickly extracted from the cranium and immersed in an ice-cold (4°C) sucrose-based artificial cerebrospinal fluid (ACSF) solution saturated with 95% O_2_ and 5% CO_2_ containing (in mM): 5, 3 KCl, 1.25 KH_2_PO_4_, 4 MgSO_4_, 20, 26 NaHCO_3_, 10 dextrose, 0.2 CaCl_2_ and 225, 219 sucrose, pH 7.3–7.4, 300–320 mosmol/kg. Coronal sections (350–400 μm thick) were performed in the same medium with a vibratome (Leica, Model VT 100S).

The rat brainstem slices were transferred to an interface-type chamber saturated with a humidified mixture of 95% O_2_–5% CO_2_. They were then perfused successively for 20 min with sucrose ACSF, then with a mixture (50–50%) of sucrose ACSF and normal ACSF (composition in mM: 124 NaCl, 5, 3 KCl, 1.25 KH_2_PO_4_, 1.3 MgSO_4_, 26 NaHCO_3_, 25, 10 dextrose and 2.4, 1.6 CaCl_2_, pH 7.3–7.4, 290–300 mosmol/kg) for 20 more minutes and finally, only with normal ACSF at 29–31°C (1 ml/min). The experiments were performed under an epifluorescence microscope (Eclipse E600FN, Nikon) where the slices were viewed at low magnification (5×) and the pools of labeled motoneurons (either with cholera-toxin or DiI) were targeted for recording.

The mice brainstem slices containing both the trigeminal main sensory nucleus (NVsnpr) and the trigeminal motor nucleus (NVmt) were transferred at room temperature to a continuously perfused holding chamber filled with normal ACSF bubbled with a mix of 95% O_2_ and 5% CO_2_. Slices were allowed to recover for at least 1 h when used to record from NVsnpr neurons, otherwise, they were directly transferred to a recording chamber perfused with ACSF (1.5 ml/min) when used to record from MNs due to their rapid deterioration.

Experiments using the Thy1-GCamp mice were performed under an epifluorescence microscope (Olympus BX50WI) equipped with an optiMOS^TM^ Scientific CMOS camera (QImaging) and a 10×-air objective for visualization and precise positioning of the pipettes in either the NVsnpr or the NVmt.

### Electroporation and Biocytin Labeling

Slices (350 μm) used for these experiments were acquired from 17 Thy1-GCaMP6f mice aged between 14 and 26 days. Electrophoresis pipettes were pulled from borosilicate glass capillaries (1.5 mm outside diameter, 1.12 mm inside diameter; World Precision Instruments, Sarasota, FL, USA) using the P-97 puller model (Sutter Instruments, Novato, CA, USA) and back-loaded with a solution of biocytin (Sigma; 1.5% in 0.5 M NaCl). A silver wire inserted inside the injection pipette was used to perform the electrophoresis. Positive pressure was applied before the descent to maintain the patency of the injection electrodes which were then guided on the surface of either the NVsnpr or the NVmt until a depression was formed. Biocytin was ejected from the pipette tip with positive rectangular pulses of current (1 μA, 7 s on, 7 s off) for 15 min. After electrophoresis, slices were kept in the chamber to recover for 2 h and then fixed for 24 h in 4% paraformaldehyde. Biocytin was revealed with streptavidin-Alexa 594 (Molecular Probes, no. S11227). The clearing was performed on the slices using the ClearT2 protocol (Kuwajima et al., 2013) to increase the imaging depth and resolution. Imaging was performed under an Olympus FluoView FV 1000 confocal microscope equipped with a 4× air and 20× water-immersion objectives (Olympus). Acquired images were processed and analyzed offline with FIJI ImageJ (NIH) and Illustrator CS4.

### Electrophysiology

Intracellular recordings were performed with brainstem slices from 35 Sprague–Dawley rats using glass microelectrodes (1.0 mm OD, 80–200 MΩ) filled with potassium acetate (3 M). Synaptic responses were evoked by electrical stimulation of the medial part of three different regions (dorsal, intermediate and ventral) of the NVsnpr using bipolar nichrome electrodes (25 μm diam) insulated except at the tip. The intensity (0.01–7 mA) and duration (0.05–0.3 ms) of the stimulus were varied to obtain optimal responses. Data were recorded using an Axoclamp 2B amplifier (Axon Instruments, Foster City, CA, USA) or a BVC-700 amplifier (Cornerstone by Dagan) through a bridge circuit and sampled at 20 kHz. Data were stored on a standard computer hard drive and analyzed using pClamp 6–8 software (Axon Instruments).

Whole-cell experiments were performed with brainstem slices from 31 Thy1-GCaMP6f mice aged between 8 and 26 days and were acquired at room temperature in a submerged recording chamber perfused with normal aCSF. Recordings of neurons from the NVsnpr and the NVmt were performed with microelectrodes pulled from borosilicate glass capillaries (1.5 mm outside diameter, 1.12 mm inside diameter; World Precision Instruments, Sarasota, FL, USA) using the P-97 puller model (Sutter Instruments, Novato, CA, USA). The microelectrodes had resistances of 5–10 MΩ and were filled with an internal solution containing (in mM) 140 potassium gluconate, 5 NaCl, 10 HEPES, 0.5 EGTA, 2 Tris ATP salt, 0.4 Tris GTP salt, pH 7.2–7.3, 280–300 mosmol/kg. Recordings were acquired in current-clamp mode using the pClamp8 software (Molecular Devices). Electrophysiological signals were amplified with the Axopatch 200B and digitized with the Digidata 1322A (Axon Instruments, Downingtown, PA, USA) and later on analyzed offline with Clampfit10.3 (Molecular Devices). Input resistance was measured using current-clamp recordings of the voltage in response to hyperpolarizing steps. Neuron viability was constantly monitored throughout the experiments with a step current-voltage protocol and only the recordings from neurons with stable resting membrane potential (RMP) of at least −45 mV and overshooting action potentials were analyzed.

Synaptic responses in trigeminal MNs were evoked by electrical stimulation (single pulse) of the NVsnpr using tungsten bipolar electrodes controlled by an Isostim A320 stimulator (WPI, Sarasota, FL, USA). Stimulation parameters were adapted for each cell to attain optimal responses except for pulse width that was kept at 0.2 ms to prevent any risk of direct stimulation of the recorded neuron. BAPTA (10 mM) was also applied locally in the dorsal NVsnpr to synaptically activate trigeminal MNs. As previously defined, we considered rhythmic bursting as a recurrent depolarization plateau over-ridden by at least three action potentials occurring at high frequency and separated by silent periods (Morquette et al., 2015). Based on the pattern and shape of both the plateau and their spikes, the bursts were classified as three different types (Ferraz-Pereira et al., 2015). Plateau potentials occurring regularly were classified as regular bursts (RB) when the spikes occurring within them are regular and perceived throughout all their extent and as adaptative bursts (AB) when the intraburst spiking is subject to an adaptation which leads to the progressive disappearance of the spikes. Finally, long-lasting plateaus occurring irregularly and with irregular spiking defined by the existence of smaller spike over-ridden plateaus were classified as irregular bursts (IB).

### Calcium Imaging

Calcium imaging in slices from 47 transgenic mice (P8–23; five of which had also served for whole-cell recordings experiments) expressing GCaMP6f under the neuronal promoter Thy1 was performed using an epifluorescence microscope (Olympus BX50WI) equipped with air (10×) and water-immersion (20× and 40×) objectives (Olympus) and an optiMOS^TM^ Scientific CMOS camera (QImaging) controlled by the open-source acquisition software Micro-Manager v1.4^1^. Acute brain slices were prepared and observed the same as for electrophysiology experiments. Excitation of GCaMP6f (470 nm) was carried out with a mercury light source (Olympus U-HGLGPS) and emission was detected through a bandpass filter (535 nm). Slices were under continuous light exposure during the recordings and images were acquired at frequencies of 5–7 Hz with no interval delay between each frame. To assess the specific topographical connectivity, we recorded calcium response of trigeminal MNs responding to electrical or pharmacological (BAPTA at 10 mM for 20 s) stimulations delivered in NVsnpr at four different and equal regions in the dorsoventral orientation. Four electrical trains (500–900 ms at 40 Hz; every 5–10 s) were delivered at the center of each location using tungsten bipolar electrodes controlled by an Isostim A320 stimulator (WPI, Sarasota, FL, USA). A blue dye that can be seen in the acquired time-lapse images was added to the BAPTA solution as a control for any possible spread of the drug outside the desired region of application in the NVsnpr.

### Drug Application

BAPTA tetrasodium salt (10 mM; Invitrogen) was locally applied with a glass pipette using the pressure pulses ejection system (Picospritzer III; Parker, Mayfield Heights, OH, USA) to induce bursting in NVsnpr neurons. In some experiments, a cocktail of blockers of excitatory and inhibitory amino-acid receptors was bath-applied with a Harvard 22 syringe pump at the following concentrations: 6-Cyano-7-nitroquinoxaline-2, 3-dione (CNQX; 10 μM; TOCRIS), D, L-2-amino-5-phosphonovaleric acid (APV; 26 μM; Sigma), GABAA Receptor Antagonist (Gabazine; 20 μM; TOCRIS).

### Imaging Analysis

Time-lapse images obtained under either the 10× air lens (0.3 N.A.) or the 20× (0.5 N.A.) and 40× (0.80 N.A.) water immersion objectives were processed and analyzed offline with FIJI ImageJ (NIH) and Excel (Microsoft). Photo-bleaching was compensated using the Bleach Correction plugin in FIJI. Changes in fluorescence intensity of responsive cells were determined by measuring for each frame the average pixel values of defined regions of interest (ROIs) traced over the cell bodies using the freehand selection tool in FIJI. The dFoverFmovie FIJI plugin was also used to help to localize lower calcium responses that may have been concealed by higher background fluorescence. An extracellular ROI was also traced near each responsive neuron for background subtraction. Calcium responses were quantified for each cell as relative changes in fluorescence intensity (ΔF) from the baseline fluorescence and were calculated in % as ΔF/F0 = (Ft − F0)/F0 where Ft is the fluorescence at a time t and F0 is the fluorescence intensity averaged over a baseline period of 1 s before the start of the stimulations. Only the neurons with changes of at least 20% and which responded either at the same delay or in synchronization with the stimulation onset were considered as “responsive cells” and included in our analysis.

### Mapping Analysis

Images acquired at 20× and 40× with traced ROIs of the responsive cells were saved in ImageJ and transferred to Adobe Illustrator to be resized and aligned over the 10× image of the nucleus of interest. For uniformity purposes, all the resulting 10× images were rotated in ImageJ so that the nuclei are vertically orientated with their dorsal and ventral poles positioned respectively at the top and bottom of the image. To produce our heatmaps, x- and y-coordinates were attributed for each responsive cell and their position was displayed in a referential normalized nucleus. To do so, the Polygon tool combined with the Bounding Rectangle option was used to trace an ROI over each nucleus to measure the size of the smallest rectangle surrounding their boundaries as well as their referential point coordinates defined as xBR and yBR. The mean width and height of rectangles measured for all the experiments were used to normalize the size of each nucleus (NVsnpr and NVmt). Afterward, we traced new ROIs over all the previously identified cells to obtain their coordinates with the 10X referential. The referential translation was used to express these coordinates in the nucleus referential using the following formula:

(xR,yR)=(x10X−xBR,y10X−yBR)

where (xR, yR) are the coordinates of the cell in the bounding rectangle referential; (x10X, y10X) are the coordinates of the cell in the 10× referential; and (xBR, yBR) are the coordinates of the bounding rectangle referential in the 10× image. These coordinates were then transferred in OriginPro 2019 (OriginLab) to create 2D Density plots and transform them into heatmaps using the Kernel Density estimation function.

### Statistics

Data are expressed as mean ± standard error (SEM) throughout the text. In all the experimental results, “*N*” represents the number of animals used while “*n*” represents the number of neurons tested unless otherwise specified. Statistical analysis was performed using the program Graphpad Sigma Stat 3.5. Paired Student’s *t*-tests were used for comparison of PSPs amplitude and frequency before and after electrical stimulation or BAPTA application. Fisher Exact tests were used for comparison of the distribution of retrogradely labeled cells or responsive neurons within NVsnpr or NVmt subdivisions. Statistical significance was defined as *P* < 0.05 in all cases.

## Results

### Electrophysiological Evidence of Connectivity Between NVsnpr and NVmt

#### Intracellular Recordings of Trigeminal MNs in Rats

**Figure 1:**
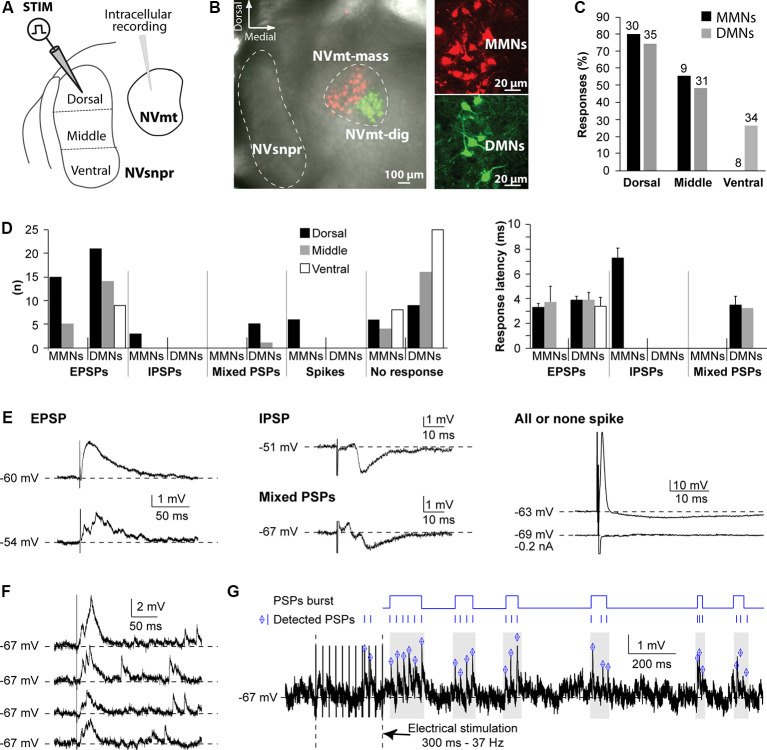
Responses elicited in trigeminal motoneurons upon stimulation of different NVsnpr divisions in rats. **(A)** Schematic illustration of the recording and stimulation (in 3 NVsnpr divisions) arrangements. **(B)** Low (left) and high (right) magnification images of MNs retrogradely labeled in NVmt after injection of Dil (red) in masseter muscles and of Cholera toxin conjugated to Alexa Fluor 488 (green) in digastric muscles. **(C)** Histograms showing the success rate of stimuli applied to each of the NVsnpr divisions for MMNs and DMNs. **(D)** Number (left) and latency (right; mean ± SEM) of each type of response elicited in the two motoneuronal pools by stimuli applied to each of the NVsnpr divisions shown in panel **(A)**. **(E)** Examples of monophasic (top) and multiphasic (bottom) EPSPs (left) and IPSPs (middle). Right: shows a typical, short latency, all or none response that results from direct activation of the recorded cell. **(F)** Long-lasting increase in the frequency of spontaneous mini EPSPs elicited by single-pulse stimulation in NVsnpr (four trials from the same stimulation site in one MN). **(G)** Top Rasters of PSPs detected in raw (bottom) traces showing clustering of PSPs that could potentially lead to burst firing in MNs after train stimulation in NVsnpr. Abbreviations: Stim, stimulation; NVsnpr, trigeminal main sensory nucleus; NVmt, trigeminal motor nucleus; NVmt-mass, masseter motoneuronal pool; NVmt-dig, digastric motoneuronal pool; MMNs, masseter motoneurons; DMNs, digastric motoneurons; EPSPs, excitatory postsynaptic potentials; IPSPs, inhibitory postsynaptic potentials; PSPs, post-synaptic potentials.

To investigate functional connections from NVsnpr to NVmt in rats, we recorded intracellularly, from different motoneuronal pools, responses elicited by stimulation of different portions of NVsnpr (as schematized in Figure 1A). In these initial studies, the emphasis was placed on the most dorsal third of NVsnpr because of earlier findings of Tsuboi et al. (2003) in the rabbit showing that NVsnpr neurons firing in phase with trigeminal MNs during fictive mastication were mostly confined to the most dorsal and medial third. Eighty recordings were made from the retrogradely labeled pool of cells (Figures 1B,C) in NVmt; 32 dorsally from masseteric MNs (MMNs) and 48 ventrally from digastric MNs (DMNs). The basic electrophysiological characteristics of MNs from the two pools did not differ remarkably from each other (RMP and input resistance = −61 ± 1 mV and 45 ± 2 MΩ and −65 ± 1 mV and 43 ± 2 MΩ for MMNs and DMNs, respectively). Electrical stimulation of dorsal NVsnpr evoked postsynaptic potentials (PSPs) in the majority of cases (19/30 of MMNs; latency 5.3 ± 2 ms and 26/35 of DMNs; latency 3.9 ± 0.3 ms) or direct activation (Figure 1E, right) in a few cases (*n* = 6 in MMNs; latency 0.6 ± 0.02 ms) presumably by current spread to their dendritic arbors which sometimes reach as far as NVsnpr. Most of these PSPs were excitatory as the ones shown in the left panel of Figure 1E (EPSPs; 15 in MMNs at a latency of 3.3 ± 0.3 ms and 21 in DMNs at a latency of 3.7 ± 1.3 ms) and many (3/16 in MMNs and 12/21 DMNs) had multiple peaks (Figure 1E, left, bottom trace) indicating either activation of other premotor neurons or perhaps responses to bursts of activity in NVsnpr. The remaining PSPs were either inhibitory, like the example shown in the middle panel (top trace) of Figure 1E (IPSPs, *n* = 3 in MMNs; latency 7.3 ± 0.8 ms) or biphasic, as illustrated in the middle panel (bottom trace) of Figure 1E (*n* = 5 in DMNs; latency 3.5 ± 0.7 ms). Stimulation of the middle portion of NVsnpr was slightly less efficient than that of the dorsal portion to elicit responses in MNs (roughly 50% success rate vs. about 66%) and evoked almost only excitatory responses (five EPSPs/9 MMNs at a latency of 3.7 ± 1.3 ms; 14 EPSPs occurring at a latency of 3.9 ± 0.6 ms and 1 biphasic PSP/31 DMNs at a latency of 3.2 ms). As was the case with EPSPs elicited by dorsal stimulation, many of these EPSPs had multiple peaks (three in MMNs and six in DMNs). Lastly, stimulation of the most ventral 3rd of NVsnpr did not elicit any response in eight MMNs and evoked nine EPSPs (at a latency of 3.4 ± 0.7 ms) in DMNs; seven of which had multiple peaks. The percentage of digastric and masseteric MNs responding to stimulation of each division of NVsnpr is depicted in Figure 1C and their number, types, and latencies are shown in Figure 1D.

In several cases (29 altogether), single-pulse stimulation in NVsnpr elicited a synaptically evoked EPSP followed by several mini EPSPs appearing at variable latencies (Figure 1F; four trials for the same stimulation). This was observed in the two populations of MNs with all stimulation sites but more frequently with dorsal stimulation (23 cases vs. 5 for the middle part and 1 for the ventral part). Repetitive stimulation (trains of 250-300 ms, 20-80 Hz) causes long lasting (from 800 ms to up to 10 s tested) occurrence of these “randomly” occurring mini EPSPs (mean frequency of 18.5 ± 2.3 Hz) that were sometimes organized into recurrent repetitive clusters (Figure 1G).

#### Whole-Cell Recordings of Trigeminal MNs in Mice

To investigate whether similar evidence of functional connectivity between NVsnpr and NVmt exist in mice, we performed whole-cell recordings of responses of trigeminal MNs (*n* = 32 from 22 mice) to electrical stimulation of NVsnpr. Trigeminal MNs had a mean input resistance of 67 ± 12 MΩ and resting membrane potentials (RMP) ranging from −45 to –74 mV (mean of −56 ± 1 mV). Nearly half of the recorded MNs (44%, *n* = 14) exhibited spontaneous tonic firing at frequencies ranging from 1 to 41 Hz with a mean of  7.5 ± 2.1 Hz. Electrophysiological characteristics of trigeminal MNs are summarized in Table 1.

**Table 1:** Electrophysiological characteristics of trigeminal MNs and NVsnpr neurons in mice. Values are mean ± SEM.

Electrophysiological	Motoneurons (*n* = 32)	NVsnpr neurons characteristics (*n* = 12)
Input resistance (MΩ)	67 ± 12	230 ± 29
RMP (mV)	−56 ± 1	−50 ± 2
Firing threshold (mV)	−42 ± 2	−40 ± 1
Spontaneous firing frequency (Hz)	7.5 ± 2.1	7.2 ± 1.8
AP amplitude (mV)	85 ± 4	60.8 ± 8.6
AP duration (ms)	1.6 ± 0.1	1.7 ± 0.3
AHP amplitude (mV)	12.4 ± 1.6	6.8 ± 1.9

*Abbreviations: MNs, motoneurons; NVsnpr, trigeminal main sensory nucleus; RMP, resting membrane potential; AP, action potential; AHP, after hyperpolarization*.

**Figure 2:**
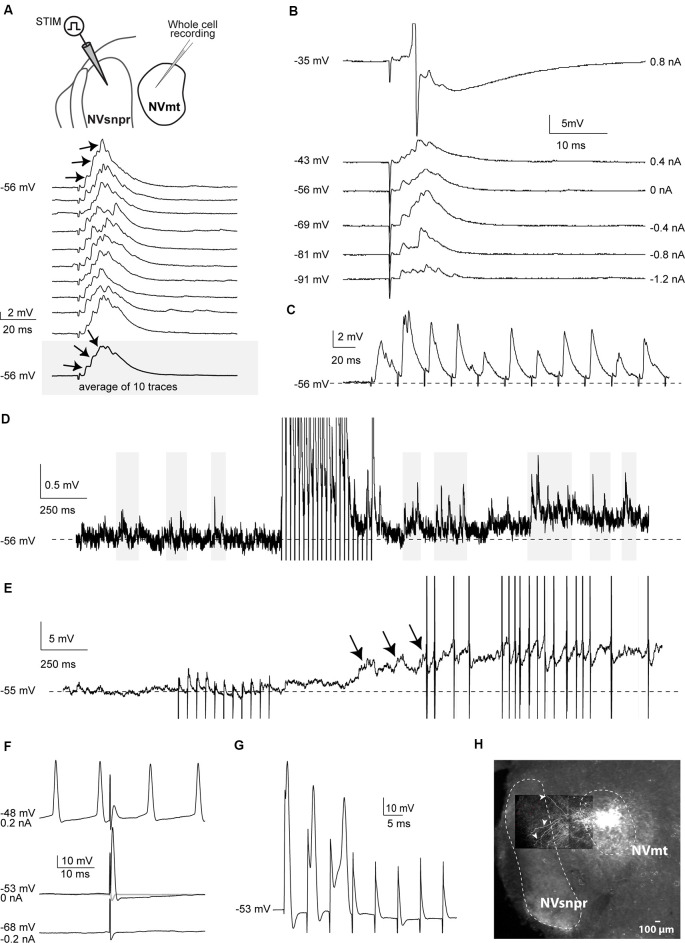
Responses elicited in trigeminal motoneurons upon stimulation of dorsal NVsnpr in mice. **(A)** top: schematic drawing of the brainstem slice preparation and the experimental conditions used. Bottom: example of a multiphasic EPSP recorded in the NVmt following electrical stimulation in the dorsal NVsnpr. Responses to 10 single pulses are shown. Inset: average trace of 10 EPSPs still shows the multiphasic component. **(B)** Depolarization does not reveal a reversal of the response. With depolarization, the stimulation elicited an action potential (Top trace, truncated). **(C)** This EPSP followed stimulation of 40 Hz. **(D)** Train of repetitive stimulations (500 ms 40 Hz) in the dorsal NVsnpr causes a long-lasting increase in the frequency of spontaneous PSPs in the recorded motoneuron. **(E)** Train of repetitive stimulations (500 ms 40 Hz) in the dorsal NVsnpr causes action potentials firing in the recorded motoneuron that seems to emerge from the summation of the increased spontaneous PSPs. **(F)** Example of a short-latency action potential (middle) elicited in the motoneuron by electrical stimulation in the dorsal NVsnpr. Hyperpolarization does not reveal an underlying postsynaptic potential (PSP; bottom) and firing preceding the stimulation causes failure (top). **(G)** High-frequency stimulation (166 Hz) reveals an inconsistency in latency and amplitude of the spike suggesting direct activation of the recorded motoneuron. **(H)** Extracellular injection of biocytin in NVmt reveals the dendritic processes of MNs extending into dorsal NVsnpr. Abbreviations: Stim, stimulation; NVsnpr, trigeminal main sensory nucleus; NVmt, trigeminal motor nucleus.

Electrical stimulation of dorsal NVsnpr evoked excitatory responses in 17 of the 32 MNs recorded. In 7 of the 17 cases (from 12 mice), these were EPSPs that occurred at a mean latency of 2.5 ± 0.2 ms and could follow frequency stimulations of 20–40 Hz suggesting that they resulted from mono- to di-synaptic connections. Their mean amplitude and duration were respectively 4.2 ± 0.7 mV and 40 ± 1 ms, and in five of these seven cases, they appeared as multiphasic and were composed of multiple overlapping synaptic events (see example in Figures 2A–C). In 5 MNs, high-frequency stimulation (40 Hz) of dorsal NVsnpr increased the frequency of spontaneous EPSPs (6.5 ± 2.3 Hz; Figure 2D). In three cases, the high-frequency stimulation also caused a sustained depolarization (6.5 ± 1.9 mV lasting 9.4 ± 3.2 s) at a mean latency of 1.0 ± 0.5 s that led (in two cases) to low frequency firing at a mean latency of 1.2 ± 0.6 s (Figure 2E). In the remaining 10 MNs, eight responded to the electrical stimulation with short-latency (1.0 ± 0.1 ms) action potentials (amplitude: 89 ± 7 mV; duration: 1.6 ± 0.3 ms; AHP amplitude: 10.6 ± 1.7 mV) that did not emerge from an underlying EPSP (Figure 2F) and display variable latency and amplitude with repetitive high-frequency stimulation (Figure 2G) indicating that they may result from direct activation of MNs through their dendrites which are known to extend far beyond the boundaries of NVmt, reaching NVsnpr (white arrows, Figure 2H), among other regions (Mong et al., 1988; Lingenhohl and Friauf, 1991).

### Anatomical Evidence of Connectivity Between NVsnpr and NVmt in Mice

**Figure 3:**
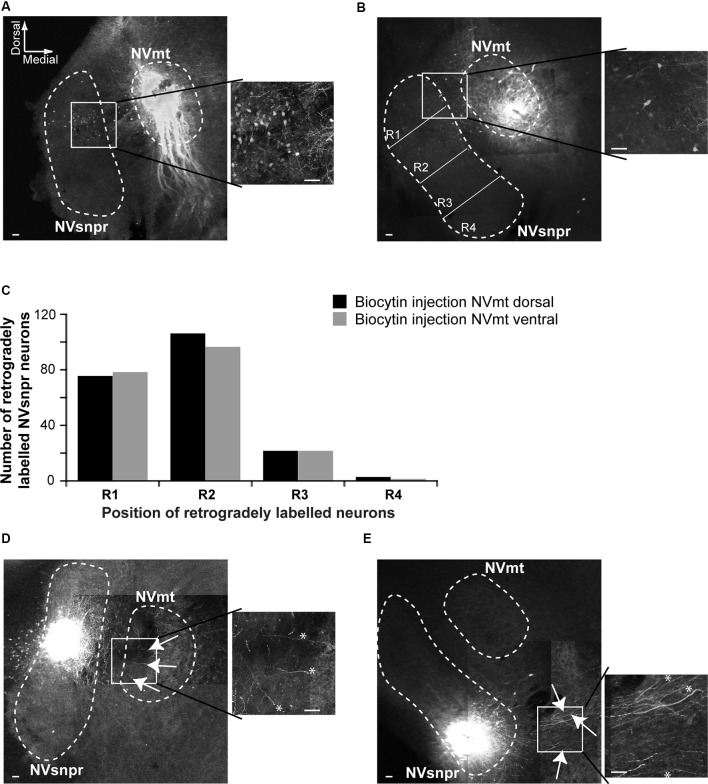
Retrograde and anterograde labeling of projections from divisions of NVsnpr to dorsal and ventral divisions of NVmt. **(A)** Left: photomicrograph showing retrogradely labeled cell bodies in the NVsnpr following extracellular injection of biocytin in the dorsal part of the NVmt. Right: enlargement of the white square in left. Calibration bars 100 μm. **(B)** Left: photomicrograph showing retrogradely labeled cell bodies in the NVsnpr following extracellular injection of biocytin in the ventral part of the NVmt. Right: enlargement of the white square in left. Calibration bars 100 μm. **(C)** Vertical bars chart reporting the distribution of retrogradely labeled cells bodies within the four subdivisions of the NVsnpr. **(D)** Left: photomicrograph showing the presence of several fibers (white arrows) in the NVmt following extracellular injection of biocytin in the dorsal part of the NVsnpr. Right: enlargement of the white square in left (*fibers pointed by the arrows in right). Calibration bars 100 μm. **(E)** Left: photomicrograph revealing the absence of fibers in the NVmt following extracellular injection of biocytin in the ventral part of the NVsnpr. Several fibers (white arrows) could be seen coursing ventrally to the nucleus. Right: enlargement of the white square in left (*fibers pointed by the arrows in right). Calibration bars 100 μm. Abbreviations: NVsnpr, trigeminal main sensory nucleus; NVmt, trigeminal motor nucleus.

To document direct projections from NVsnpr to NVmt in mice, and to examine if these projections follow a topographic organization, we made injections of biocytin into the dorsal (NVmt-D) and ventral (NVmt-V) divisions of NVmt for retrograde labeling of NVsnpr neurons (Figures 3A,B) or into the dorsal and ventral divisions of NVsnpr for anterograde labeling to NVmt (Figures 3D,E). NVsnpr was arbitrarily divided into four regions (R1, R2, R3, and R4; see Figure 3B) to count retrogradely labeled neurons as shown in insets of Figures 3A,B. Both injections sites in NVmt yielded roughly similar numbers (injections in NVmt-D yielded a total of 207 cells in five slices from five mice, and injections in NVmt-V labeled a total of 196 cells in three slices from three mice) and distribution patterns of retrogradely labeled cells among NVsnpr divisions (injection in NVmt-D vs. NVmt-V: R1/75 vs. 78 cells, R2/109 vs. 96 cells, R3/21 vs. 21 cells and R4/2 vs. 1 cell; Figure 3C; Fisher Exact Test, *P* = 1, R1+R2 vs. R3+R4). Thus, projections to both dorsal and ventral NVmt originated almost exclusively from the dorsal $\frac{3}{4}$ of NVsnpr, with the highest number of retrogradely labeled neurons in R1 and R2, much fewer in R3 and nearly none in R4 (Figure 3C).

To further support these results, we injected biocytin into the dorsal (R1+R2, six slices from six mice) and ventral (R3+R4, four slices from three mice) divisions of NVsnpr to assess the direction of their axonal projections to NVmt. In three of the six slices tested, injections of biocytin in dorsal NVsnpr resulted in labeling of thin fibers projecting to or terminating in NVmt (white arrows and asterisks in Figure 3D), whereas injections in ventral NVsnpr resulted in labeling of thin fibers mostly in a region ventral to NVmt (white arrows and asterisks in Figure 3E) also known as the parvocellular reticular formation (PCRt).

**Figure 4:**
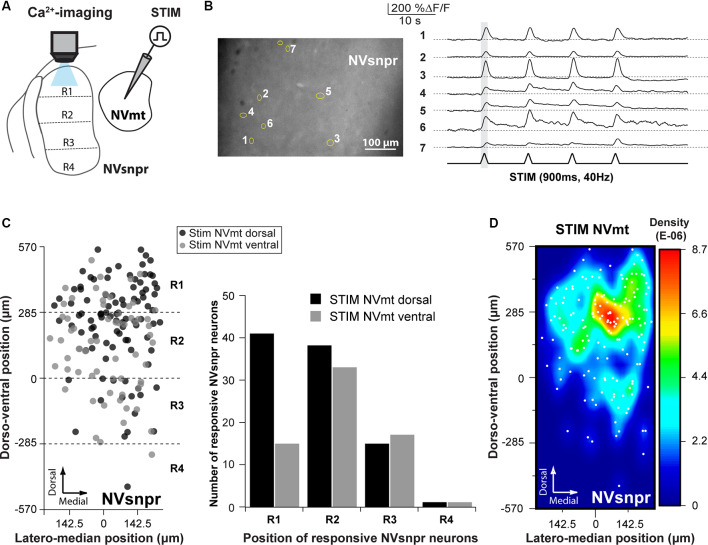
Distribution of NVsnpr neurons activated by stimulation of NVmt. **(A)** Schematic drawing of the brainstem slice preparation and the experimental conditions used. **(B)** Photomicrograph showing the position of responsive cells in NVsnpr following electrical stimulation of dorsal NVmt and their synchronous Ca^2+^ responses. Gray vertical line indicates the length of the train of stimulation. The bottom trace shows a stimulus artifact. **(C)** Distribution of cells responding to stimulation of both NVmt-D and NVmt-V throughout NVsnpr (Left) and count of the number of cells per division in NVsnpr (Right). **(D)** Heatmap representing the density of responsive neurons in NVsnpr following stimulation of NVmt-D and NVmt-V pooled together. Abbreviations: Stim, stimulation; NVsnpr, trigeminal main sensory nucleus; NVmt, trigeminal motor nucleus.

**Figure 5:**
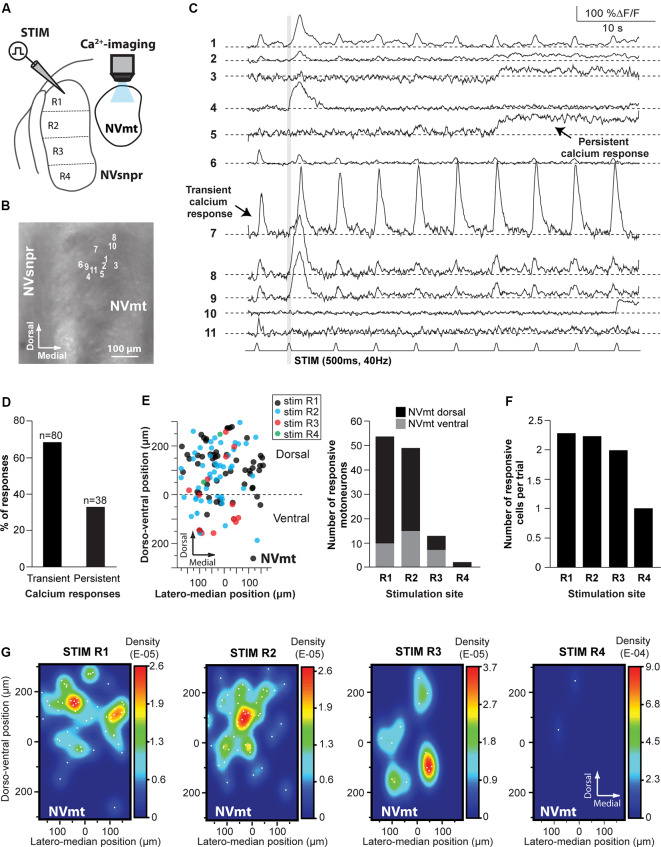
Distribution of motoneurons activated by stimulation of different divisions of NVsnpr. **(A)** Schematic drawing of the brainstem slice preparation and the experimental conditions used. **(B)** Photomicrograph showing the position of the responsive cells in NVmt following electrical stimulation of the dorsal NVsnpr and their Ca^2+^-responses in panel **(C)**. Some showed a transient response at every stimulation pulse (e.g., trace 7), while others had persistent responses (e.g., trace 5) or only one large transient response with or without a few much smaller ones afterward. Gray vertical line indicates the length of the train of stimulation. **(D)** Percentage of NVsnpr cells presenting transient vs. persistent responses. **(E)** Left: distribution of all responsive motoneurons according to the site of electrical stimulation in NVsnpr. Right: vertical bars chart reporting the number of responsive motoneurons according to the site of electrical stimulation in the NVsnpr. **(F)** Vertical bars chart reporting the number of responsive motoneurons per trial according to the site of electrical stimulation in the NVsnpr. **(G)** Heatmaps illustrating the location of responsive motoneurons within the NVmt according to the site of electrical stimulation in the NVsnpr. Abbreviations: Stim, stimulation; NVsnpr, trigeminal main sensory nucleus; NVmt, trigeminal motor nucleus.

However, retrograde and anterograde labeling can always result from the uptake of the tracer from passing by fibers. Thus, to further document direct connectivity between the two nuclei, we recorded Ca^2+^ and electrophysiological changes elicited in neurons of one of the two nuclei upon electrical stimulation of the other (as schematized in Figures 4A, 5A).

### Functional Evidence of Connectivity Between NVsnpr and NVmt Revealed by Calcium Imaging

Electrical stimulation (500–900 ms train, 40 Hz) in either NVmt-D (*N* = 15) or NVmt-V (*N* = 13) elicited synchronized transient calcium responses (261 ± 20%ΔF/F0 lasting 2.2 ± 0.1 s; Figure 4B) in 161 NVsnpr neurons (in 23 slices from 12 of 14 mice tested). These neurons were distributed essentially throughout the dorsal $\frac{3}{4}$ of the nucleus (Figure 4C) with the highest density located mainly in its dorsomedial area (Figure 4D). Stimulation of NVmt-D elicited a higher number of responses in the most dorsal part of NVsnpr (R1) relative to stimulation of NVmt-V and both divisions elicited a comparable number of responses in R2 and R3. However, the distributions of the responsive NVsnpr neurons within R1 vs. R2+R3 in response to both stimulation sites in NVmt were not statistically different (Fisher Exact Test, *P* = 1).

Electrical stimulation (500–900 ms train, 40 Hz) in four different regions of the NVsnpr elicited a monophasic increase of intracellular calcium (as in Figure 5C) in 118 MNs (in a total of 60 slices from 35 mice. R1: 54 MNs in 18 of 30 mice, R2: 49 MNs in 10 of 15 mice, R3: 13 MNs in three of five mice and R4: 2 MNs in two of five mice; see Figure 5E), throughout the dorsal and ventral NVmt. These were mostly (68% of cases) transient responses (160 ± 15% ΔF/F0 lasting 3.4 ± 0.3 s; *n* = 80/118) characterized by a fast rise and a slow decay that out lasted the stimulation train (Figures 5C,D). The remaining 32% (38 out of 118 MNs) exhibited persistent calcium responses (87 ± 16% ΔF/F0) with a minimum duration ranging between 3 and 55 s (Figures 5C,D). To ensure that responses observed in NVmt did not result from direct activation of motoneuronal dendrites that sometimes reach NVsnpr, a cocktail was used in three animals to block glutamatergic and GABAergic receptors [6-Cyano-7-nitroquinoxaline-2,3-dione (CNQX) 10 μM; D, L-2-amino-5-phosphonovaleric acid (APV) 26 μM; Gabazine; 20 μM]. Bath-application of these blockers abolished calcium responses in four out of six (67%) responding MNs (not shown). In general, each (trial) electrical stimulation performed in NVsnpr elicited few responses in the NVmt (mean cells/trial: 2.29 for R1, 2.25 for R2, 2.00 for R3 and 1.00 for R4; Figure 5F). The highest number of responsive cells per stimulation was obtained with R1 which in one case activated a cluster of 11 MNs in the dorsolateral quadrant (see MNs positions in Figure 5B) of NVmt (an example is shown in Figure 5C). This region also holds the highest concentration and density of responsive cells which may reflect the highest level of connectivity with NVsnpr (Figures 5E,G). Stimulation of the first three regions (R1–R3, black, blue, and red circles in Figure 5E) of the NVsnpr generated nearly all the calcium responses elicited in trigeminal MNs. In five mice, only two MNs located in the dorsolateral quadrant responded to the stimulation of R4 (R4, green circles in Figures 5E,G, right panel).

In general, activated MNs were observed throughout NVmt but were more concentrated in certain areas depending on the stimulated site. Stimulation of the dorsal NVsnpr resulted mainly in the activation of MNs located dorsally [R1: 44 of 54 MNs (82%) and R2: 35 of 49 MNs (71%)]. However, there was a slight lateral and ventral shift of the cell clusters when R2 was stimulated compared to R1 where the clusters were mostly located dorsally (Figure 5G, 2 left panels). Of the four NVsnpr areas, stimulation of R2, which is also the area with the highest connectivity to NVmt (Figures 3C, 4D), produced the highest ratio (67% of tested mice) and number (4.9/animal) of calcium responses in NVmt. Fewer stimulation attempts were made in R3, but the number of elicited responses/trial was similar to that obtained with stimulation of more dorsal areas (Figure 5F). However, these tended to be located more ventrally in NVmt (red circles in Figures 5E,G, 2nd from right). A slight trend of connectivity pattern emerges from a comparison of the heatmaps of R1, R2 and R3 with the position of regions of the higher density of activated neurons in NVmt shifting in the same direction in the dorsoventral axis as the displacement of the stimulation in NVsnpr (Figure 5G). However, despite the obvious trend of connectivity between both nuclei, the distributions of the responsive MNs in response to stimulation of the dorsal vs. the ventral part of NVsnpr were not statistically different (Fisher Exact Test, *P* = 1).

We then questioned the pattern of activity elicited in MNs when NVsnpr neurons fire rhythmically.

### Induction of Rhythmic Firing in NVsnpr Neurons and Transmission to MNs

#### BAPTA-Induced Bursting in NVsnpr Neurons

**Figure 6:**
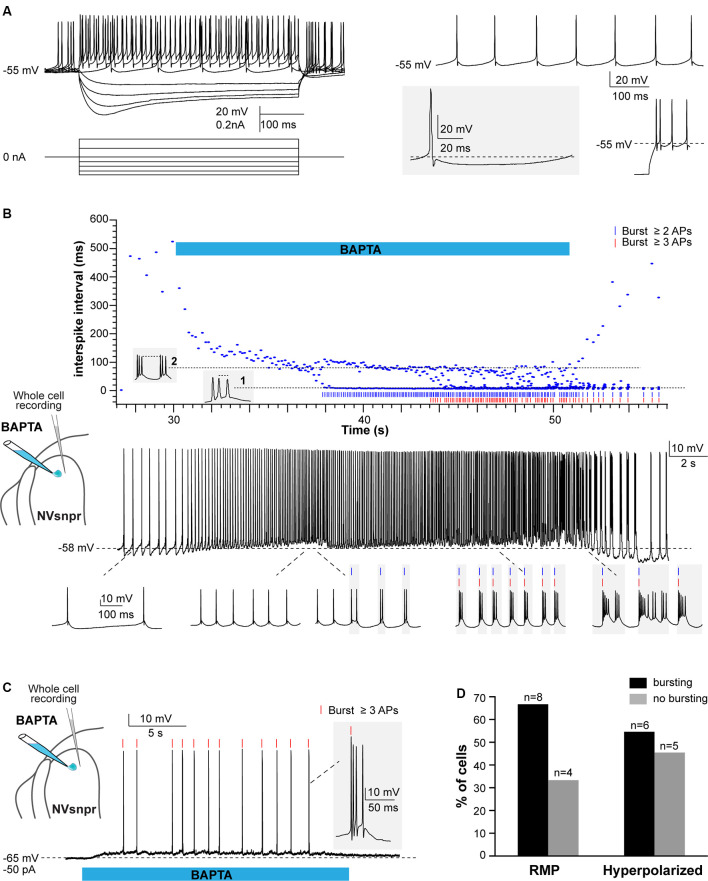
Local applications of BAPTA induce rhythmic burst firing in NVsnpr neurons. **(A)** Left: membrane responses of an NVsnpr neuron (top traces) to injections of depolarizing and hyperpolarizing current pulses (bottom traces). Right top: spontaneous activity recorded in an NVsnpr neuron (inset bottom enlargement showing the biphasic after hyperpolarization (AHP). Right bottom: example of the rebound firing observed at the offset of membrane hyperpolarization. **(B)** Top: scatter plot of the inter-spike interval prior, along with and after local application of BAPTA at the NVsnpr neuron resting potential. The pattern of firing gradually progresses from tonic to bursting along the course of BAPTA application (Bottom traces). **(C)** Upon membrane hyperpolarization, the BAPTA application directly causes burst firing (inset) in the NVsnpr neuron. **(D)** Percentage of NVsnpr cells in which bursting was induced by local BAPTA applications at their RMP or upon imposed hyperpolarization. Abbreviations: NVsnpr, trigeminal main sensory nucleus; APs, action potentials; RMP, resting membrane potential.

To induce rhythmic firing in NVsnpr we used local extracellular applications of BAPTA (10 mM) in NVsnpr of Thy1-GCaMP6f mice (*N* = 9, P10–19) as previously done in rats (Morquette et al., 2015). The basic electrophysiological characteristics of 12 dorsal NVsnpr neurons recorded for this purpose are summarized in Table 1. Ten of these had spontaneous activity as in Figure 6A (right, top trace) and a depolarizing sag upon hyperpolarization (Figure 6A, left, top traces) with nine of them displaying rebound spiking upon the termination of the current injection (see Figure 6A, right, bottom trace).

In 8 of the 12 neurons recorded, BAPTA applications at the RMP induced rhythmic bursting (Figures 6B,D, bottom traces), but in seven of these, there was first a depolarization (6.1 ± 1.6 mV) and/or an increase in firing frequency for those that were spontaneously active, before the switch in firing pattern indicated by the abrupt drop of the inter-spike interval (ISI; occurring at a latency of 2.4 ± 0.8 s in the example shown in Figure 6B (top) and its oscillation between two levels; one reflecting the intra-burst frequency and one the inter-burst frequency. Recurrent bursting produced a hyperpolarization (7.2 ± 1.6 mV) in five cases lowering the neurons membrane potential to approximately −62 ± 3 mV. When tested at more hyperpolarized potentials (−61 ± 2 mV), BAPTA induced a depolarization (*n* = 4; 5.4 ± 1.7 mV at a latency of 2.8 ± 1.0 s) followed by a tonic firing (*n* = 2; 9 ± 4 Hz at a latency of 10.1 ± 1.2 s) or bursting (*n* = 6; at a latency of 7.0 ± 2.3 s) in 8 out of 11 tested NVsnpr neurons (see Figures 6C,D). The bursts elicited at the RMP consisted of three different types, the regular bursts (RB; *n* = 4; 50%), the adaptative bursts (AB; *n* = 1) and the irregular bursts (IB; *n* = 3; 38%). RB appeared at −60 ± 2 mV and were represented by recurrent short plateaus (10.3 ± 2.6 mV, lasting 384 ± 137 ms, 0.9 ± 0.4 Hz) with over-riding action potentials occurring regularly at a frequency of 69.3 ± 15.9 Hz. AB was elicited in only one neuron and was characterized by recurrent plateaus (0.4 Hz) of larger amplitudes (30.8 mV) and durations (1,200 ms) that set off at a potential of −62 mV. Finally, the three remaining neurons displayed IB characterized by an irregular occurrence (0.9 ± 0.4 Hz) of plateaus that set-off at an average potential of −48 ± 6 mV and that could be further subdivided into smaller plateaus. The bursts elicited upon membrane hyperpolarisation (occurring at −59 ± 2 mV with a mean latency of 7.0 ± 2.3 s) were mainly RB (*n* = 3; plateaus of 9.4 ± 1.6 mV lasting 86 ± 22 ms, at 1.0 ± 0.2 Hz) with two IB (1.0 ± 0.1 Hz) and only one AB (plateaus of 30.9 mV lasting 1,700 ms, at 0.3 Hz). Interestingly, the majority of the bursts with irregular intra-burst spiking (two AB and three of five IB) were elicited in mice younger than P12 (P10 and P11) which coincides with the age of emergence of the first masticatory movements in the rat (Westneat and Hall, 1992).

#### Calcium Imaging of NVsnpr Neurons

**Figure 7:**
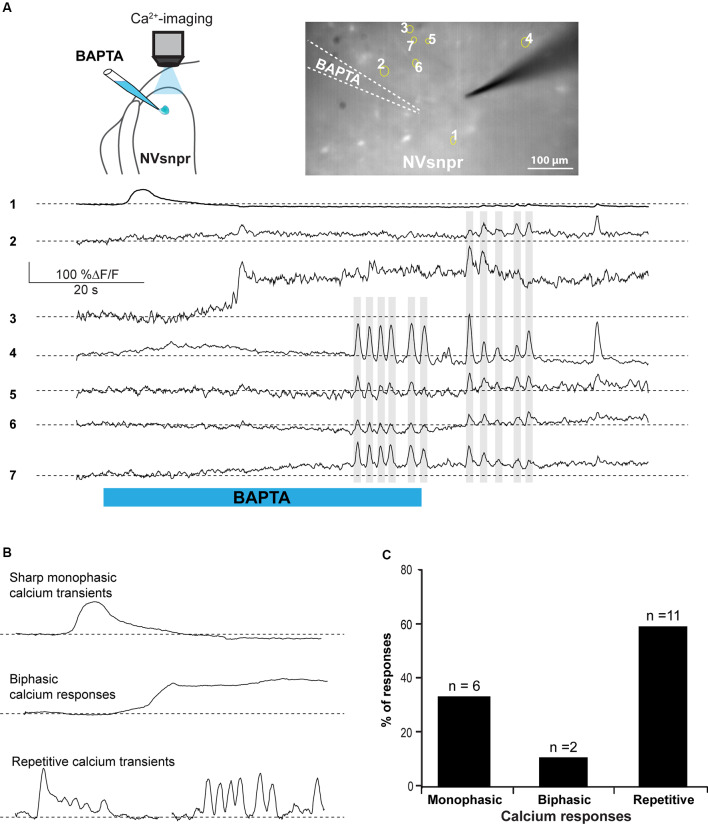
BAPTA applications near NVsnpr neurons induce transient and recurrent Ca^2+^-responses that can sometimes occur synchronously in nearby neurons. **(A)** Right top: schematic drawing of the brainstem slice preparation and the experimental conditions used. Left top: photomicrograph showing the position of the responsive cells in the NVsnpr following local BAPTA application. Bottom: calcium responses of several NVsnpr neurons following the BAPTA application. Gray lines crossing several traces emphasize synchronicity between recurrent events occurring in different cells. **(B)** Illustration of the three main types of calcium responses observed in NVsnpr neurons following local application of BAPTA. **(C)** Vertical bars chart reporting the percentage (and number) of types of calcium responses observed in NVsnpr neurons following local applications of BAPTA. Abbreviation: NVsnpr, trigeminal main sensory nucleus.

Whole-cell recordings in the current-clamp configuration allow for real-time monitoring of electrophysiological activity and manipulation of the membrane potential of the recorded cell but do not provide information about the number, synchrony, and distribution of activated cells. Calcium imaging of NVsnpr neurons in Thy1-GCaMP6f mice was used for that purpose. Local BAPTA (10 mM, in two mice) applications in two slices from these mice elicited mainly transient calcium responses in 19 NVsnpr neurons located near the site of application with a maximum radius of activation of approximately 420 μm (distance between the tip of the BAPTA pipette and the farthest responsive NVsnpr neuron). Responses occurred at variable latencies (mean of 17.3 ± 4.0 s) depending on the distance from the pipette tip. NVsnpr neurons responded with either single or repetitive Ca^2+^ increases to single BAPTA applications. Single Ca^2+^ responses (187 ± 87% ΔF/F0) most often (*n* = 6) consisted of an initial fast rising phase followed by a slow decay period (Figure 7A, trace 1, and Figure 7B top trace) and lasted 8.6 ± 2.8 s. In two additional cases, the Ca^2+^-response (396 ± 10 5% ΔF/F0) did not decay after the initial rise and persisted as a long-lasting plateau until the end of the BAPTA application (as in Figure 7B middle trace). In the 11 other neurons (58%; Figure 7C), repetitive Ca^2+^ transients peaks (110 ± 12% ΔF/F0 lasting 4.2 ± 1.6 s) were observed (Figure 7A, traces 4–7) occurring at a mean frequency of 0.38 ± 0.04 Hz. In five of these cases, the repetitive Ca^2+^ transients peaks overrode a plateau-like calcium transient as in the examples shown in Figure 7A, trace 3, and Figure 7B (left, bottom trace). Very interestingly, many of these recurrent events occurred synchronously in several adjacent cells (see gray lines in Figure 7A).

#### Trigeminal MNs Respond to BAPTA-Induced Bursting in NVsnpr

##### Whole-Cell Recordings of MNs

**Figure 8:**
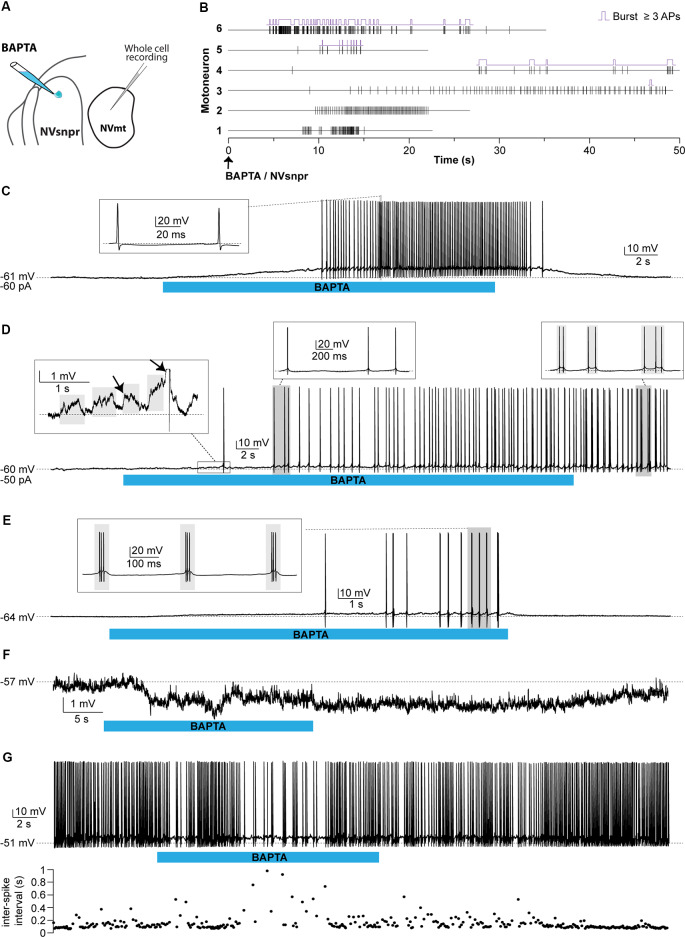
Effects of local BAPTA applications in NVsnpr on motoneuronal firing. **(A)** Schematic drawing of the brainstem slice preparation and the experimental conditions used. **(B)** Raster plots of firing induced in six MNs that were silent before applications of BAPTA in NVsnpr. Traces are aligned on the BAPTA application. The firing patterns elicited in the MNs were single spikes as in panel **(C)**, that sometimes emerged from the summation of subthreshold PSPs (left inset in panel **D**), and progressed to doublets as in panel **(D)** (right inset). **(E)** BAPTA application in the dorsal NVsnpr also sometimes caused burst firing (left, inset) in the recorded motoneuron as in this case. **(F)** Long-lasting hyperpolarization or decrease in firing frequency **(G)** as indicated by the raw trace (top) or raster plot (bottom) of the inter-spike intervals were observed at a mean latency of 5.2 ± 2.8 s in two MNs. Abbreviations: NVsnpr, trigeminal main sensory nucleus; NVmt, trigeminal motor nucleus; APs, action potentials; RMP, resting membrane potential.

**Figure 9:**
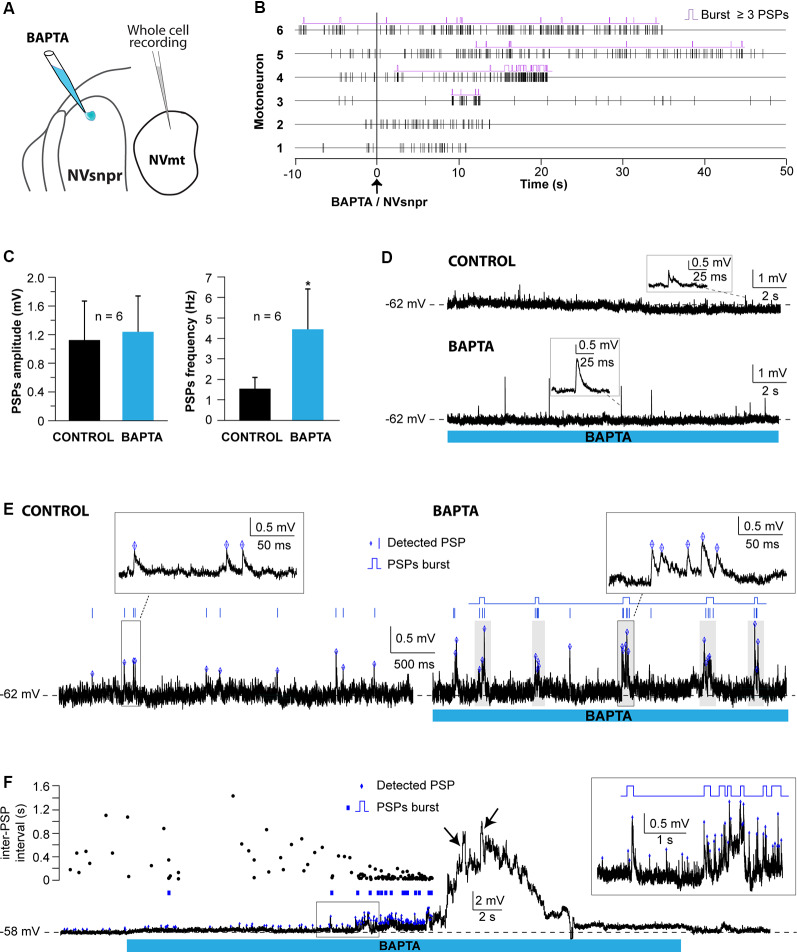
Effects of local BAPTA applications in NVsnpr on subthreshold motoneuronal activity. **(A)** Schematic drawing of the brainstem slice preparation and the experimental conditions used. **(B)** Raster plots of detected PSPs in six MNs before and after the application of BAPTA in NVsnpr. Traces are aligned on the BAPTA application. **(C)** Effect of the BAPTA application on the amplitude (left) and frequency (right) of these PSPs. Data are mean ± SEM; *P < 0.05, paired Student’s *t*-test. **(D)** Example of a case where spontaneous PSPs recorded in the MN were of greater amplitude after BAPTA application in NVsnpr. **(E)** Example of a case where BAPTA application in NVsnpr induced rhythmic (right) clusters of PSPs (right inset) in the recorded MN. **(F)** BAPTA application in the dorsal NVsnpr elicited repetitive depolarizations caused by the summation of spontaneous PSPs (inset) in the recorded motoneuron. The raster plot above the raw trace shows a decrease of the inter-PSP intervals a few seconds before these large summation events. Abbreviations: NVsnpr, trigeminal main sensory nucleus; NVmt, trigeminal motor nucleus; PSPs, post-synaptic potentials.

Responses of trigeminal MNs to BAPTA applications in dorsal NVsnpr were first assessed electrophysiologically with recordings from 20 MNs distributed across the NVmt (as schematized in Figures 8A, 9A). No discernible effects were seen in 6 of the 20 recorded neurons following BAPTA applications in the NVsnpr. In the remaining 14, a clear effect was seen on the MNs spiking ability (*n* = 7) or its subthreshold activity (*n* = 7). In the former seven cases, BAPTA in NVsnpr induced firing in previously silent neurons (*n* = 6) and reduced the firing frequency in a single spontaneously active neuron (from 8.8 to 3.7 Hz as evidenced by the increased inter-spike interval upon BAPTA (bottom scatter plot; Figure 8G). Firing induced in the six other MNs upon BAPTA application in the NVsnpr occurred at a mean latency of 7.1 ± 0.8 s and is shown as raster plots aligned to the start of BAPTA application in Figure 8B. This spiking was preceded by depolarization (2.8 ± 1.6 mV occurring at a latency of 5.8 ± 2.2 s) that lead to either tonic firing of single-action potentials (Figure 8C; *n* = 2), doublets (Figure 8D; *n* = 1) of action potentials or rhythmic bursts at a frequency of 0.9 ± 0.4 Hz (Figure 8E; *n* = 3).

In the seven cases, where BAPTA applications in NVsnpr appeared to affect the MNs subthreshold activity, six were excitatory and one was inhibitory as above. The latter appeared as a long-lasting hyperpolarization (as in Figure 8F; 3.8 mV, lasting 49 s). The excitatory effects on the six other MNs are presented as raster plots of detected PSPs, aligned to the start of the BAPTA application in Figure 9B. BAPTA seemed to cause an increase in the amplitude of the spontaneous PSPs, as in the example shown in Figure 9D, but this effect was not significant when all the neurons were pooled (Figure 9C; 1.1 ± 0.5 mV vs. 1.2 ± 0.5 mV, paired *t*-test, *P* = 0.09). However, BAPTA application in the NVsnpr significantly increased the frequency of the spontaneous EPSPs (Figure 9C; 1.5 ± 0.5 Hz vs. 4.4 ± 2 Hz, paired Student’s *t*-test, *P* = 0.02) and caused in many cases (*n* = 4) appearance of rhythmic clusters of PSPs (as in Figure 9E), that may be indicative of a direct rhythmic input that does not reach firing threshold. BAPTA also caused the appearance of recurrent depolarizations (as in Figure 9F; see arrows; 4.6 ± 0.9 mV lasting 1.0 ± 0.3 s) characterized by synchronized groups of high amplitude spontaneous EPSPs (*n* = 4). This type of response appeared at a mean latency of 11.5 ± 6.9 s with an average frequency of 1.1 ± 0.2 Hz and lasted for approximately 43 ± 10 s. In one MN, the appearance of spontaneous EPSPs combined with an increase of their frequency (0–40 Hz) following BAPTA application can be observed preceding the occurrence of such repetitive depolarizations (see Figure 9F inset). In two out of four MNs, these repetitive depolarizations summated and lead to a transient depolarization of longer duration (4.5 ± 2.6 s) and greater amplitude (7.1 ± 2.5 mV). In the two remaining MNs, the repetitive depolarizations were overridden by APs (singlets or duplets as in the inset of Figure 8D) suggesting that each one of them may have resulted from bursting NVsnpr neurons near the BAPTA pipette.

These excitatory effects of BAPTA applied in the NVsnpr were more frequent in MNs at hyperpolarized holding potentials (*n* = 7, −65 ± 1 mV) compared to when it was tested at RMP (*n* = 4, −60 ± 3 mV). For instance, 4 out of 11 MNs exhibited rhythmic bursts or doublets (RB type as in Figure 8E) when tested at hyperpolarized potentials, with only two of them occurring when tested at their RMP.

##### Calcium Imaging of MNs

**Figure 10:**
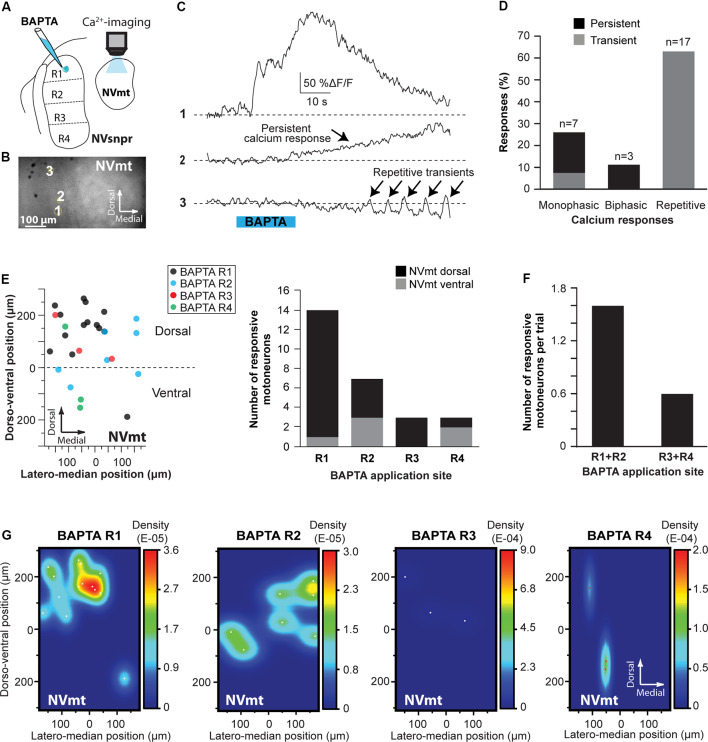
Distribution and types of Ca^2+^-responses elicited in NVmt by local applications of BAPTA in NVsnpr. **(A)** Schematic drawing of the brainstem slice preparation and the experimental conditions used. **(B)** Photomicrograph showing the position of the responsive cells in the NVmt following BAPTA application in the dorsal NVsnpr. **(C)** Types of Ca^2+^-responses recorded in three different MNs after BAPTA applications in the dorsal NVsnpr. **(D)** Vertical bars chart representing the percentage of cells displaying each type of Ca^2+^-response. **(E)** Left: distribution of all the responsive motoneurons according to the site of BAPTA application in the dorsal NVsnpr. Right: vertical bars chart reporting the number of responsive MNs according to the site of BAPTA application in the dorsal NVsnpr. **(F)** Vertical bars chart reporting the number of responsive motoneurons per trial according to the site of BAPTA application in the dorsal NVsnpr. **(G)** Heatmaps illustrating the location of the responsive motoneurons within the NVmt according to the site of BAPTA application in NVsnpr. Abbreviations: NVsnpr, trigeminal main sensory nucleus; NVmt, trigeminal motor nucleus.

In calcium imaging experiments (as schematized in Figure 10A), local BAPTA application in the four regions of NVsnpr (R1, R2, R3, and R4) elicited calcium responses (as the examples shown in Figure 10C; see MNs positions in Figure 10B) in 27 MNs (in 12 of 25 mice) located mainly in the dorsal NVmt (*n* = 21) with only six cells responding ventrally (see Figures 10E–G). Dorsal BAPTA applications in NVsnpr induced more calcium responses in NVmt (1.4/animal) than ventral applications (0.6/animal; Figure 10F) with the highest ratio obtained by R2 (1.8/animal). Although, no clear distribution pattern of the responding MNs in NVmt was observed across stimulation sites in NVsnpr, there is no overlap between regions activated by R1 and R2 (Figure 10G).

The Ca^2+^-responses elicited by BAPTA applications were either single or repetitive increases that occurred at a mean latency of 13.9 ± 2.2 s (Figures 10C,D). Single events were either transient (*n* = 2; 66 ± 16%ΔF/F0 for 22.4 ± 9.0 s) or persistent and lasted as long (*n* = 5; 106 ± 23%ΔF/F0) or longer than the BAPTA application (*n* = 3; 210 ± 70%ΔF/F0). In the 17 remaining cases, BAPTA applications in NVsnpr produced repeated calcium transients in MNs (83 ± 27%ΔF/F0 for 2.9 ± 0.5 s, see trace 3 in Figure 10C) at a mean frequency of 0.4 ± 0.1 Hz and in 11 of these, the repetitive calcium transients overrode long plateaus calcium transients of 11.3 ± 2.9 s.

## Discussion

The present results provide new evidence of functional connectivity between the NVmt and the NVsnpr, thought to form the rhythmogenic core of the masticatory CPG (Athanassiadis et al., 2005a,b; Brocard et al., 2006; Kolta et al., 2007, 2010; Morquette et al., 2015). Our results indicate that neurons projecting to different parts of the NVmt are located in the dorsal $\frac{3}{4}$ region of NVsnpr (R1, R2, and R3). Electrical stimulation of the dorsal NVsnpr induced multiphasic excitatory synaptic responses in trigeminal MNs while BAPTA applications induced rhythmic firing in NVsnpr that translated in rhythmic activities in NVmt, further supporting the hypothesis that NVsnpr may drive the masticatory rhythmic motor pattern. Anatomical and functional mapping of projections from NVsnpr to NVmt suggests that dorsal NVsnpr projects preferentially to dorsal NVmt whereas intermediate parts of NVsnpr project more ventro-medially in NVmt. However, the most ventral part of NVsnpr does not project to NVmt. This study confirms and expands earlier findings (Li et al., 1995, 1996; Stanek et al., 2014) by exploring the physiological nature and functional topography of the connectivity between NVsnpr and NVmt that was demonstrated in the past with neuroanatomical techniques.

### Connectivity

#### Anatomical Evidence

Our anatomical experiments suggest that NVsnpr neurons projecting to both the dorsal or ventral NVmt emanate mostly from the dorsal three-quarter region (R1, R2, and R3) of NVsnpr. In both cases, the highest number and density of retrogradely labeled NVsnpr neurons were found constricted within the dorsal half (R1 and R2) with very few observed in R3. Moreover, injections of biocytin within NVsnpr resulted in labeled fibers originating exclusively from the dorsal region and terminating both in the DL and VM divisions of NVmt further supporting this connectivity evidence. This is consistent with previous anatomical studies demonstrating in both rats (Li et al., 1995, 1996) and mice (Stanek et al., 2014) that the majority of NVsnpr projections to NVmt arises from neurons located in the dorsal division.

Although labeled fibers originating from the ventral NVsnpr did not project to the NVmt, we observed multiple fibers terminating into PCRt, the region directly ventral to it. PCRt, a nucleus recently associated with hunger-driven mastication and feeding modulation (Nakamura and Nakamura, 2018), is also known to form direct synaptic contact with both jaw-closing and jaw-opening MNs (Mogoseanu et al., 1993; Kolta et al., 2000; McDavid et al., 2006; Yoshida et al., 2009). In the same region but closer to the ventral division of the NVsnpr is the group k nucleus formed by small neurons organized in a column (Mukerji et al., 2009). This nucleus contains the tensor tympani MNs and is believed to be responsible for the attenuation of sounds generated during the mastication process (Mukerji et al., 2010). Furthermore, there is additional evidence showing in the rabbit that masticatory muscles receive direct innervation from the group k nucleus (Donga et al., 1992; Saad et al., 1997, 1999). However caution should be used in the interpretation of these results since retrograde labeling may partly result from the uptake by fibers of passage inside NVmt, and because of the small size of the VM division and its proximity to the DL, the specific labeling of jaw-opening pre-MNs becomes even more challenging.

#### Electrophysiological Evidence

In the present study, nearly half of the trigeminal MNs evoked short-latency EPSPs following electrical stimulation of the dorsal NVsnpr suggesting a monosynaptic connection with NVmt. Moreover, these synaptic responses were for the majority multiphasic in nature which may have resulted from a convergence of monosynaptic glutamatergic inputs from multiple pre-MNs stimulated in NVsnpr or possibly from activation of polysynaptic pathways. Many anatomical studies demonstrated that the region in the lateral reticular formation surrounding NVmt [peritrigeminal area (PeriV) and PCRt] is home of the largest population of trigeminal pre-MNs (Mizuno et al., 1983; Landgren et al., 1986; Chandler et al., 1990; Stanek et al., 2014) which are known to form a complex network of interconnected excitatory and inhibitory neurons that could shape the final masticatory motor output (Kolta, 1997; Bourque and Kolta, 2001). NVsnpr has been shown to share reciprocal connectivity with both PeriV and PCRt, further supporting the implication of polysynaptic pathways originating from NVsnpr (Shammah-Lagnado et al., 1992; Kolta, 1997; Yoshida et al., 1998; Kolta et al., 2000; Athanassiadis et al., 2005b). An alternative explanation of such responses would be the convergence of multiple synaptic inputs received through the extensive dendritic arborization of trigeminal jaw-closing MNs known to extend beyond the boundaries of NVmt and even reaching inside the NVsnpr (Mong et al., 1988; Lingenhohl and Friauf, 1991). This type of connectivity is not unusual for trigeminal MNs where it was demonstrated in earlier electron microscopic studies that axon terminals of most trigeminal pre-MNs surrounding the NVmt make synaptic contact more often with the dendrites than with the soma of MNs (Mizuno et al., 1978, 1983; Mogoseanu et al., 1993).

### Motoneurons Respond to High-Frequency Stimulation in NVsnpr

Stimulation of the dorsal NVsnpr at 40 Hz, a frequency close to the natural output of the sensory afferents during mastication (Trulsson and Johansson, 2002), evoked intracellular calcium transients in MNs located mainly in the DL division of NVmt. The persistence of these responses beyond the stimulation trains could be partially reflecting the slow calcium dissociation kinetics of GCaMP but is likely to also reflect real slow decay of the intracellular calcium concentration (Yoshida et al., 2001; Chen et al., 2013). In some cases, a long-lasting response was evoked in MNs following several electrical trains. In calcium imaging, these responses were characterized by a slow and persistent rise of calcium which could probably represent a gradual and slow increase of the spike frequency while in whole-cell recordings the response represented a depolarization leading to an increasing spike discharge. This interpretation is based on evidence showing that GCaMP is mainly sensitive to spike discharge and not to smaller membrane potential changes (Grienberger and Konnerth, 2012; Chen et al., 2013; Badura et al., 2014; Helmchen and Tank, 2015). The slope and duration of the rising phase of the recorded calcium transients would then correspond to the spike frequency and firing duration, respectively (Yoshida et al., 2001). This comes from the fact that calcium transients from internal stores are not necessarily correlated with changes in membrane potential (Nakajima and Baker, 2018). Moreover, an increase in the frequency of spontaneous EPSPs was also induced, indicating a possible synaptic facilitation effect caused by the high-frequency stimulations. Alternately, the long-lasting responses and putative facilitation effects may also result from activation of the polysynaptic pathways referred to above which have been described as forming strong redundant connections (Bourque and Kolta, 2001) or from activation of astrocytic networks. Our previous work has shown that 40 Hz stimulation of inputs to NVsnpr activates NVsnpr astrocytes that release a Ca^2+^-binding protein (S100β) that decreases extracellular Ca^2+^ concentration (Morquette et al., 2015). This in turn potentiates a Na^+^ persistent current leading to burst-firing in NVsnpr neurons. This process occurs over hundreds to thousands of milliseconds and may explain the longer latency and duration of some of the responses elicited in MNs.

### Topography

Biocytin injections and calcium imaging, used to investigate the specific organization of the projections from NVsnpr to the NVmt, revealed that the dorsal part of NVsnpr projects mostly to the DL division containing the jaw-closing MNs. The electrical stimulation of NVmt revealed that the dorsomedial part of NVsnpr, which coincides with the region known to innervate the masseter MNs, is the main region projecting to trigeminal MNs (Appenteng and Girdlestone, 1987). Interestingly, this region is also known to arbor the majority of neurons with intrinsic bursting properties (Athanassiadis et al., 2005b). Previous retrograde HRP labeling studies performed in rats and mice revealed the myotopical arrangement of jaw-closing and jaw-opening MNs within NVmt: (1) those innervating the masseter muscle are located in the lateral part of the DL division; (2) those innervating the temporal muscle are located in the dorsomedial part of the DL division; (3) those innervating the medial and lateral pterygoid muscles are located in the most ventral part of the NVmt; and (4) those innervating the mylohyoid and the anterior belly of the digastric muscle are located in the VM division of NVmt (Chen et al., 1988; Terashima et al., 1994; Setsu et al., 2001).

In our experiments, the masseter region was activated by the dorsal NVsnpr with R2 resulting in a larger activated region in the lateral part of NVmt. On the other hand, the temporal region was innervated most exclusively by R1 suggesting a smaller and more distinct representation of the temporal pre-MNs within NVsnpr compared to those of the masseter which cover the whole dorsal region. Interestingly, the jaw-closing representations within NVmt are known to be proportional to the innervated muscle size (Maeda et al., 1993; Watson et al., 2012) and this seems to be also preserved within the NVsnpr. Although most NVsnpr regions (R1, R2, and R3) evoked responses in the ventral NVmt, our results revealed a dorso-ventral tendency where the activation ratio in the ventral NVmt was increasing and the recruited regions were shifting ventrally through NVmt. R3 was the only region exhibiting an exclusive ventral innervation of NVmt and activated the highest density of MNs within VM which suggests that it might preferentially innervate the lateral and medial pterygoid and the jaw-opening MNs. These observations are in line with an anterograde study conducted on the rat reporting that trigeminal pre-MNs located in the dorsal NVsnpr make axon contact preferentially to jaw-closing MNs while those located in the intermediate part of NVsnpr make axon contact primarily to jaw-opening MNs (Li et al., 1995).

### BAPTA-Induced Rhythmic Activity in NVsnpr

We showed in previous studies performed in rats that bursting could be elicited in NVsnpr neurons with local BAPTA application and that this rhythmic discharge is I_NaP_-dependent as it can be abolished by pharmacological blockade of the current (Brocard et al., 2006; Morquette et al., 2015). Therefore, BAPTA could be used as a burst-inducing stimulus in NVsnpr to experiment rhythm transmission toward NVmt. However, despite being currently the most commonly used animal model for fundamental research, there are still no reports investigating this mechanism in mice. We successfully evoked bursting with BAPTA in 67% of the recorded NVsnpr neurons at a membrane potential within the activation range of I_NaP_ (between −65 and −50 mV; Crill, 1996). In most cases, a depolarization followed by spiking preceded the bursts which occurred at frequencies ranging from 0.4 to 0.9 Hz.

The spatiotemporal activity pattern induced within the BAPTA application site in NVsnpr appeared as a circular wave with sequential activation of NVsnpr neurons. Not only NVsnpr neurons responded at variable latencies, but also the patterns evoked were not always rhythmic as expected. Indeed, BAPTA evoked single Ca^2+^ events in 42% and repetitive calcium transients in 58% of neurons which correspond approximately to the proportion of bursting response at RMP (67%) obtained in whole-cell recordings. Thus, it might suggest that those who responded with only one transient could be too depolarized to activate I_NaP_ knowing that BAPTA caused a depolarization in 60% of the recorded NVsnpr. Recurrent transients occurred at frequencies comparable to the bursts recorded electrophysiologically in whole-cell patch configuration but the duration of the peak was longer in calcium imaging which could be due to the slow calcium decay phase of GCaMP6f (Chen et al., 2013; Li et al., 2019). Earlier reports have demonstrated that neural rhythmic patterns are detectable with calcium imaging techniques and that their sensitivity highly depends on the decay kinetics of the calcium indicator (Yoshida et al., 2001; Lin et al., 2007; Chen et al., 2013). If the calcium decay phase is longer than the inter-spike interval, a summation would occur and therefore, multiple action potentials would be represented by a long-lasting calcium transient. Thus, the long-lasting singular transients evoked in NVsnpr neurons with BAPTA could perhaps result from trains of action potentials while repetitive calcium transients would represent rhythmic bursting.

### Motoneurons Respond to BAPTA-Induced Bursting in NVsnpr

BAPTA application in NVsnpr successfully induced rhythmic bursting in MNs at a membrane potential within the I_NaP_ activation range. Another rhythmic activity characterized by recurrent plateau potentials was evoked in MNs. It was shown in guinea pigs that trigeminal motoneurons have bistable membrane properties mediated by I_NaP_ and L-type Ca^2+^ currents required for the production of such plateau potentials (Hsiao et al., 1998). In our study, these plateau potentials occurred at frequencies resembling those evoked in NVsnpr in patch-clamp and were, in 50% of the cases, overridden by action potentials. Therefore, we think that these recurrent depolarizations might represent the bursts evoked in NVsnpr neurons with BAPTA.

In calcium imaging, BAPTA evoked rhythmic calcium transients occurring at similar frequencies than those obtained in NVsnpr neurons. Moreover, responding MNs upon BAPTA application were found mainly within the DL division of NVmt. These MNs responded mostly when BAPTA was applied within the dorsal NVsnpr. This result is consistent with our findings stipulating that the jaw-closing MNs receive most of their inputs from the dorsal NVsnpr where both the masseter and temporal pre-MNs are localized.

## Conclusion

Mastication is the first step of nutrition, but has also been linked to many other functions including systemic health and cognitive performance (Yamamoto and Hirayama, 2001; Kamiya et al., 2016; Lin, 2018). In animal studies (reviewed in Weijenberg et al., 2019) factors impacting mastication, such as molar loss or abrasion or soft food diets have been associated with poorer spatial memory and learning ability as well as decreased proliferation and differentiation of newborn neurons in the hippocampus. Both measures increase when molars are restored with crowns or when hard food is given back. In humans, mastication is compromised in several pathologies [e.g., Down syndrome (Faulks et al., 2008), Parkinson’s (Ribeiro et al., 2017), Alzheimer’s (Campos et al., 2017) and Huntington’s diseases (Trejo et al., 2004)], and in the elderly where masticatory dysfunction is increasingly considered as an unrecognized risk factor contributing to the development of cognitive impairment (Tada and Miura, 2017). Thus, a better understanding of the circuitry and mechanisms responsible for mastication is important to help elaborate strategies to modify masticatory efficiency to slow the rate of cognitive decline and improve cognitive health during aging. Although the pattern of masticatory movements may vary across species, the mechanisms and general organization of the circuits generating them are likely to be similar. Human studies report an increase in blood flow in NVsnpr during mastication (Viggiano et al., 2015), but cannot further address how the activity is triggered in this nucleus and how it is relayed to the motoneurons controlling the masticatory muscles. The results reported here suggest that projections from NVsnpr to NVmt are similarly organized in rats and mice with the dorsolateral region of NVmt receiving inputs from the dorsal NVsnpr (R1 and R2), and the ventromedial region receiving mostly inputs from R2 and R3. Knowing that different areas of NVsnpr receive topographically organized sensory inputs may help devise intervention strategies for reeducation. From a functional perspective, NVsnpr neurons were also shown to have the capacity to drive rhythmic activity in trigeminal MNs, thus reinforcing their potential role in masticatory rhythmogenesis (Athanassiadis et al., 2005a,b; Brocard et al., 2006; Kolta et al., 2007, 2010; Kadala et al., 2015; Morquette et al., 2015).

## Data Availability Statement

The datasets generated for this study are available on request to the corresponding author.

## Ethics Statement

The animal study was reviewed and approved by Comité de déontologie de l’expérimentation sur les animaux/Université de Montréal.

## Author Contributions

MS, DV, and AK designed the research. MS, IA, and SO performed the research. MS, DV, and AK analyzed the data and wrote the article.

## Conflict of Interest

The authors declare that the research was conducted in the absence of any commercial or financial relationships that could be construed as a potential conflict of interest.

## Footnotes

^1^http://www.micro-manager.org
